# Incorporation of Aliphatic Proline Residues into Recombinantly Produced Insulin

**DOI:** 10.1021/acschembio.3c00561

**Published:** 2023-11-14

**Authors:** Stephanie L. Breunig, Janine C. Quijano, Cecile Donohue, Amy Henrickson, Borries Demeler, Hsun Teresa Ku, David A. Tirrell

**Affiliations:** 1 Division of Chemistry and Chemical Engineering, California Institute of Technology, Pasadena, California 91125, United States; 2 Department of Translational Research and Cellular Therapeutics, Diabetes and Metabolism Research Institute, Beckman Institute City of Hope, Duarte, California 91010, United States; 3 Department of Chemistry and Biochemistry, University of Lethbridge, Lethbridge, Alberta T1K 3M4, Canada; 4 Department of Chemistry and Biochemistry, University of Montana, Missoula, Montana 59801, United States; 5 Irell & Manella Graduate School of Biological Science, City of Hope, Duarte, California 91010, United States

## Abstract

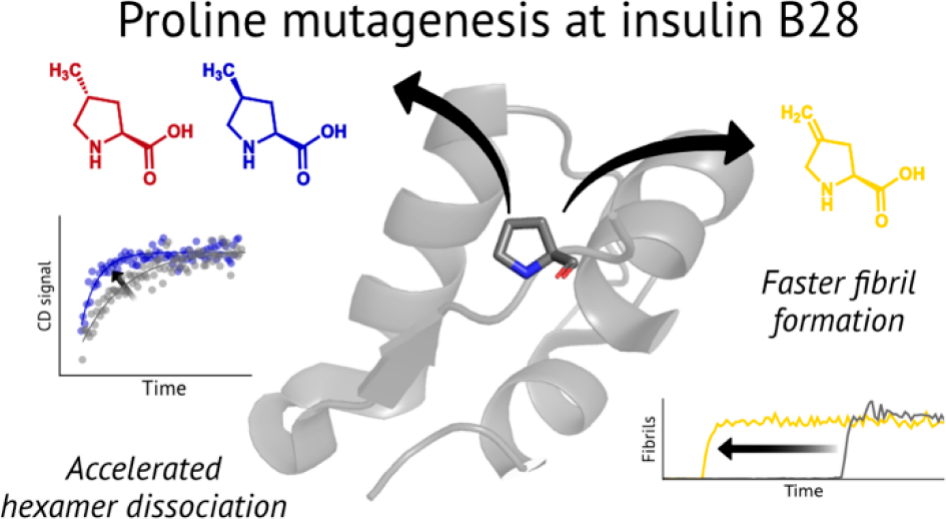

Analogs of proline can be used to expand the chemical space about the residue while maintaining its uniquely restricted conformational space. Here, we demonstrate the incorporation of 4*R*-methylproline, 4*S*-methylproline, and 4-methyleneproline into recombinant insulin expressed in *Escherichia coli*. These modified proline residues, introduced at position B28, change the biophysical properties of insulin: Incorporation of 4-methyleneproline at B28 accelerates fibril formation, while 4-methylation speeds dissociation from the pharmaceutically formulated hexamer. This work expands the scope of proline analogs amenable to incorporation into recombinant proteins and demonstrates how noncanonical amino acid mutagenesis can be used to engineer the therapeutically relevant properties of protein drugs.

## Introduction

Proline is unique among the canonical amino acids: the cyclic pyrrolidine side chain restricts the conformational space accessible to the residue. Replacing proline with any of the proteinogenic amino acids through standard mutagenesis approaches necessarily grants greater conformational freedom. Alternatively, noncanonical proline (ncPro) residues expand the chemical space about proline while maintaining a pyrrolidine (or pyrrolidine-like) side chain. Because the conformational preferences of many proline analogs are known,^1,2^ ncPro residues are useful as probes of conformational effects on protein behavior. Proline analogs have enabled investigators to elucidate the importance of a key proline *cis*-*trans* isomerization event in 5HT_3_ receptor opening,^3^ modify the properties of elastin-like proteins,^4−6^ determine the molecular origins of collagen stability,^1,7^ and probe the role of *cis*-*trans* isomerization in β2-microglobulin fibrillation.^8,9^ Although Chatterjee and co-workers have reported progress in the development of orthogonal prolyl-tRNA synthetase/tRNA pairs,^10^ to date, only residue-specific (rather than site-specific) incorporation approaches have been successful in introducing proline analogs into recombinant proteins.^11^ Using *E. coli* as an expression host, these approaches have allowed efficient incorporation of various 3- and 4-functionalized proline residues and those with modified ring sizes and compositions.^12^

**Figure 1:**
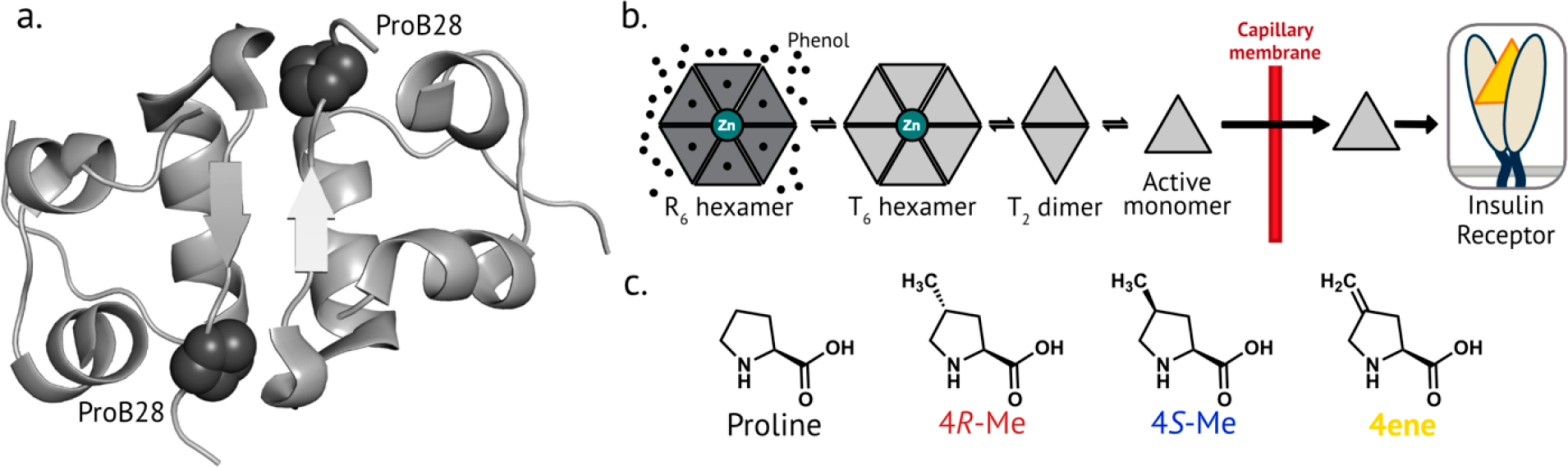
Proline mutagenesis at position B28 of human insulin. a. Crystal structure of insulin (PDB 1MSO), highlighting ProB28 located at the dimer interface. b. Simplified scheme of insulin dissociation after injection. Insulin exists as a hexamer in the R state in the presence of zinc and phenolic ligands such as in the pharmaceutical formulation. After injection, insulin dissociates into lower-order oligomeric species that can diffuse more easily across the capillary membrane, enter the bloodstream, and bind to the insulin receptor. c. The structure of proline and the aliphatic proline analogs used in this study.

Insulin (Figure 1a) is a 5.8 kDa peptide hormone normally released from the pancreatic β cells in response to elevated levels of blood glucose. Its binding to the insulin receptor induces intracellular responses that ultimately lower blood glucose concentrations.^13^ Diabetes mellitus is a result of dysfunctional insulin signaling, either through an impaired ability to produce and secrete insulin (type 1) or through insulin resistance (type 2). Subcutaneous injection of exogenous insulin is a common strategy in diabetes treatment, especially for individuals with type 1 diabetes. The recombinant production of insulin^14^ has significantly advanced diabetes treatment by enabling both its production at scale and the creation of modified insulins with desirable properties through standard mutagenesis and chemical modification approaches.^15−18^

To mimic the insulin-action profile of a healthy pancreas, two broad classes of insulin analogs have been developed: long-acting (or basal) insulins and fast-acting insulins (FAIs).^19^ Long-acting variants recapitulate the lower levels of insulin secretion that maintain metabolism in an anabolic state. Conversely, FAIs aim to mimic the transient increases in insulin concentration stimulated by elevated blood glucose after a meal. Typical insulin replacement therapy relies on a combination of regular basal insulin treatments and FAI injections before meals.

Many FDA-approved insulin variants have been engineered by altering the amino acid sequence in ways that cause pronounced pharmacokinetic effects.^20^ Notably, insulin aspart^17,21^ (NovoLog, marketed by Novo Nordisk) and insulin lispro^15,22^ (Humalog, Eli Lilly) both involve changes to ProB28, a key residue^15^ near the C-terminus of the B-chain (Figure 1a). Insulin aspart is achieved by the single point mutation ProB28Asp, while insulin lispro contains an inversion of ProB28 and LysB29; both changes destabilize insulin oligomers. Because the rate-limiting step for insulin absorption into the bloodstream is dissociation of oligomer to monomer^23^ (Figure 1b), these changes accelerate insulin’s onset of action. Insulins are also prone to chemical and physical denaturation,^24−26^ processes slowed by the formation of protective oligomers. As a result, insulin production and distribution require a cold chain,^27^ and maintaining protein stability in continuous subcutaneous insulin infusion pumps is challenging.^28^

Intrigued by the role that ProB28 plays in insulin biophysics, we recently introduced a series of ncPro residues at position B28 of human insulin.^29,30^ Because mature insulin contains one proline, a residue-specific replacement approach results in site-specific proline replacement without the need for an orthogonal aminoacyl-tRNA synthetase/tRNA pair. These efforts, which focused on proline analogs known to incorporate well into recombinant proteins in *E. coli*,^5^ illustrated how proline mutagenesis of insulin can be used to tune its biophysical characteristics.^29,30^

Here, we demonstrate the efficient incorporation of three new aliphatic proline residues (4*R*-methylproline, 4*R*-Me; 4*S*-methylproline, 4*S*-Me; and 4-methyleneproline, 4ene; Figure 1c) at position B28 of recombinantly produced insulin; these insulin variants will be referred to as ins-4*R*-Me, ins-4*S*-Me, and ins-4ene, respectively. We find that these modifications alter insulin behavior: replacement of ProB28 with 4ene speeds fibril formation, while 4-methylation accelerates hexamer dissociation without affecting stability against physical denaturation. This work expands the range of proline analogs that can be incorporated into recombinant proteins in *E. coli*. It also demonstrates how small molecular changes introduced through noncanonical amino acid mutagenesis can be used to probe and engineer the therapeutically relevant properties of protein drugs.

## Results and Discussion

### Aliphatic Proline Residues Are Accepted by the *E. coli* Translational Machinery

**Figure 2:**
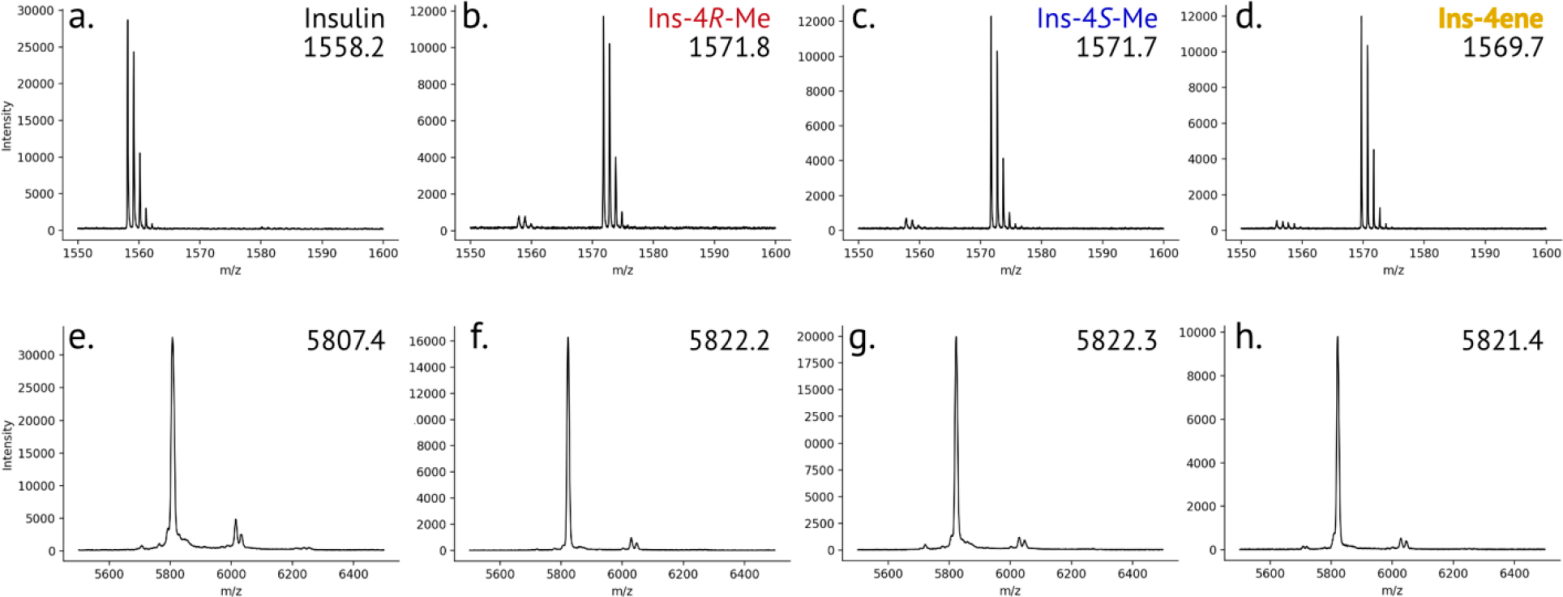
Mass spectrometry of insulin variants. a-d. Characterization of proline analog incorporation. The solubilized inclusion body (containing proinsulin) after expression in medium supplemented with proline (a), 4R-Me (b), 4S-Me (c), or 4ene (d) was digested with Glu-C and analyzed by MALDI-TOF MS. The peptide that contains position B28 of mature insulin is ^50^RGFFYT**P**KTRRE (expected *m*/*z* = 1557.8). e-h. MALDI-TOF characterization of mature and purified insulin variants: human insulin (e), ins-4*R*-Me (f), ins-4*S*-Me (g), and Ins-4ene (h). The peaks at *m*/*z* ∼6050 correspond to adducts of the sinapic acid matrix.

To identify an expanded set of ncPro residues accepted by the *E. coli* translational machinery, we expressed proinsulin (a precursor to insulin) under conditions that favor ncPro incorporation.^12^ We monitored ncPro replacement by proinsulin expression and mass spectrometry and noted a range of incorporation efficiencies for the 15 commercially available proline analogs tested (Figure S1 and Table S1). Notably, the aliphatic proline residues 4*R*-Me, 4*S*-Me, and 4ene led to high levels of proinsulin expression and good (∼90%) incorporation efficiencies under the optimized conditions (Figure 2a-d, Table S2, Table S3). We replaced ProB28 of human insulin with each of these three aliphatic proline analogs (Figure 2e-h) and determined the resulting effects on insulin behavior.

### Proline Analogs Do Not Affect Insulin Secondary Structure or Bioactivity

**Figure 3:**
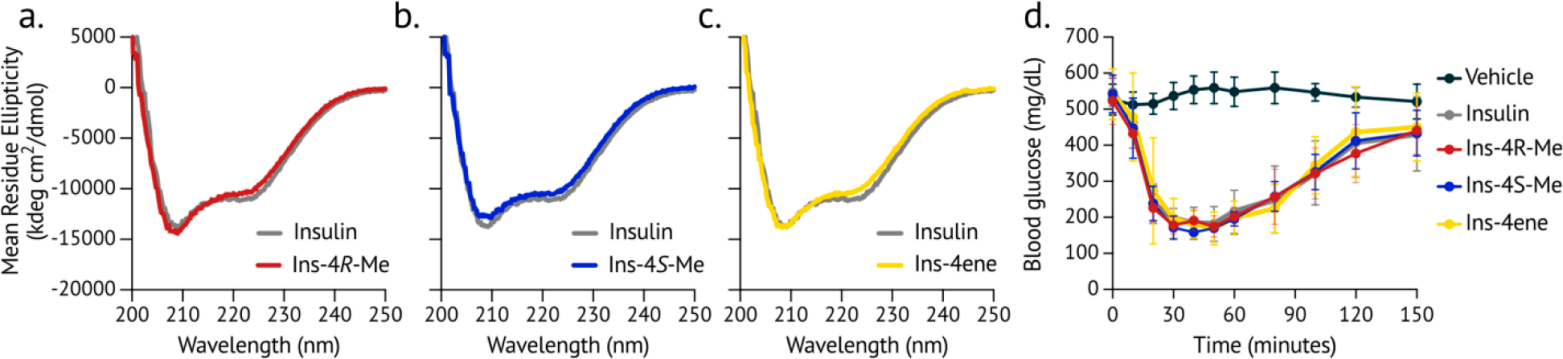
Circular dichroism spectroscopy and bioactivity of insulin variants. a-c. Far-UV circular dichroism spectra of insulin and insulin variants (60 μM in 100 mM phosphate buffer, pH 8.0). The spectrum of each insulin variant is overlaid with that of human insulin (gray). d. Insulins were injected subcutaneously into diabetic mice, and blood glucose was measured over time after injection.

The secondary structure of each insulin variant was assessed by circular dichroism (CD) spectroscopy (Figure 3a-c; Table S4). The CD spectrum of insulin is sensitive to the state of oligomerization of the protein: for monomeric insulins, the ratio of negative ellipticities at 208 and 222 nm is increased compared to that of the insulin dimer.^31^ The far-UV CD spectrum for each variant closely matched that of human insulin at 60 μM, suggesting that proline replacement has not significantly perturbed the secondary structure or dimer formation under these conditions.

To validate biological activity *in vivo*, insulins were formulated with zinc and phenolic ligands and injected subcutaneously into diabetic mice; blood glucose was monitored over 2.5 h. Rodent models can assess insulin activity but cannot distinguish differences in time to onset of action for human insulin and fast-acting analogs.^32^ Because the C-terminus of the B-chain does not interact with the insulin receptor,^33,34^ we did not expect modification of ProB28 to affect bioactivity. As anticipated, all of the insulin variants reduced the blood glucose in diabetic mice (Figure 3d).

### 4-Methylation of ProB28 Speeds Hexamer Dissociation

**Figure 4:**
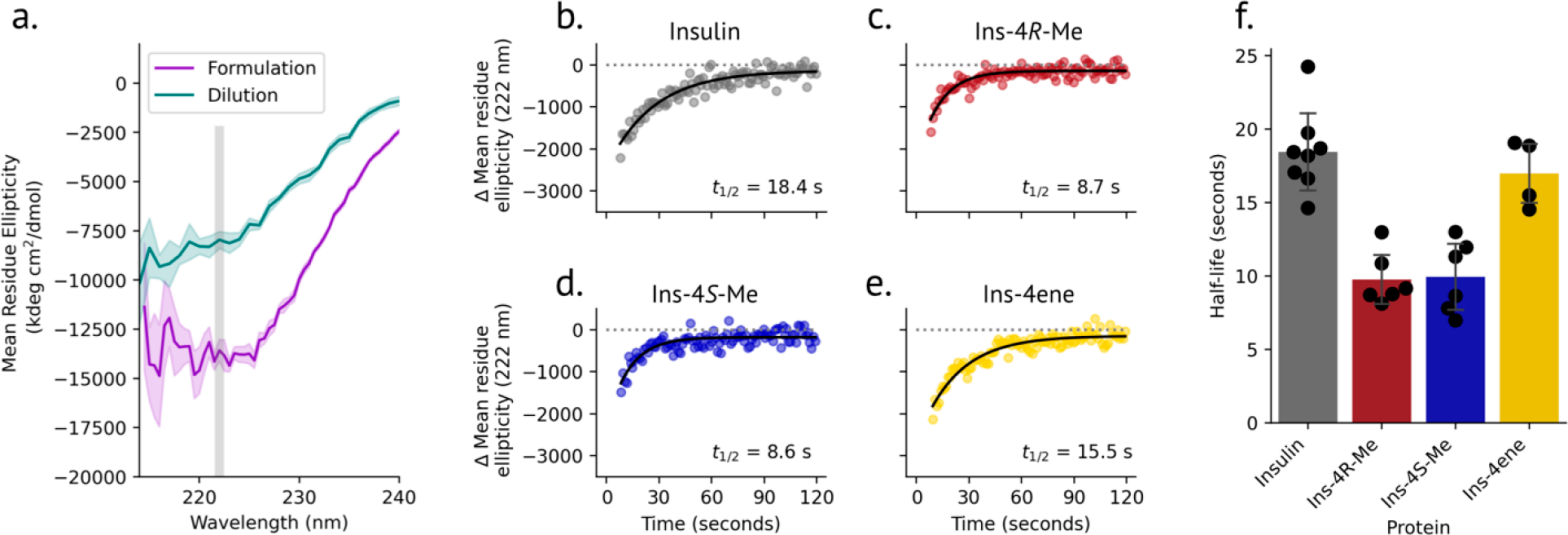
Hexamer dissociation kinetics of insulin variants. a. Equilibrium CD spectra of insulin before and after dilution. To measure the dissociation kinetics, the decrease in negative ellipticity at 222 nm was monitored over time after dilution. b-e. Representative dissociation plots for insulin (b), ins-4*R*-Me (c), ins-4*S*-Me (d), and ins-4ene (e). Each dilution experiment was fit to a monoexponential function, and the half-life for each displayed replicate is indicated. f. Summary of dissociation half-life values.

Increased negative ellipticity at 222 nm in the CD spectrum of insulin is a signature of oligomerization.^31,35^ Adopting a method reported by Gast and co-workers,^36^ we measured the rate of dissociation of insulin variants to the monomer state. Insulins were formulated under conditions that mimic the pharmaceutical formulation (600 μM insulin, 25 mM *m*-cresol, 250 μM ZnCl_2_) and favor the R_6_ hexamer state.^37^ Dissociation was monitored by tracking the mean residue ellipticity at 222 nm over time after 150-fold dilution into ligand-free buffer (Figure 4a). CD spectra after dilution are distinct from that of chemically denatured insulin (Figure S3, Figure S4), confirming that changes in the CD signal are not a result of denaturation. While ins-4ene dissociated at a rate similar to that of human insulin (t_1/2_ = 17.0 ± 2.3 and 18.4 ± 2.8 s, respectively), dissociation of ins-4*R*-Me (t_1/2_ = 9.8 ± 1.8 s) and ins-4*S*-Me (t_1/2_ = 9.9 ± 2.5 s) was accelerated under these conditions (Figure 4b-f, Table S4).

It seems likely that steric effects play a role in accelerating the dissociation of ins-4*R*-Me and ins-4*S*-Me. The carbon atoms of 4*R*- and 4*S*-methyl substituents installed at ProB28 in published structures of the R_6_ insulin hexamer are in close proximity (2.2 and 2.3 Å, respectively) to backbone carbonyl oxygen atoms of the adjacent monomer (Figure S5a); these distances are smaller than the sum of the van der Waals radii of the respective atoms (1.70 Å for carbon and 1.52 Å for oxygen).^38^ Similar interactions are observed in the T_6_ hexamer (Figure S5b). The resulting steric clashes may act to destabilize the hexamer and accelerate dissociation.

**Figure 5:**
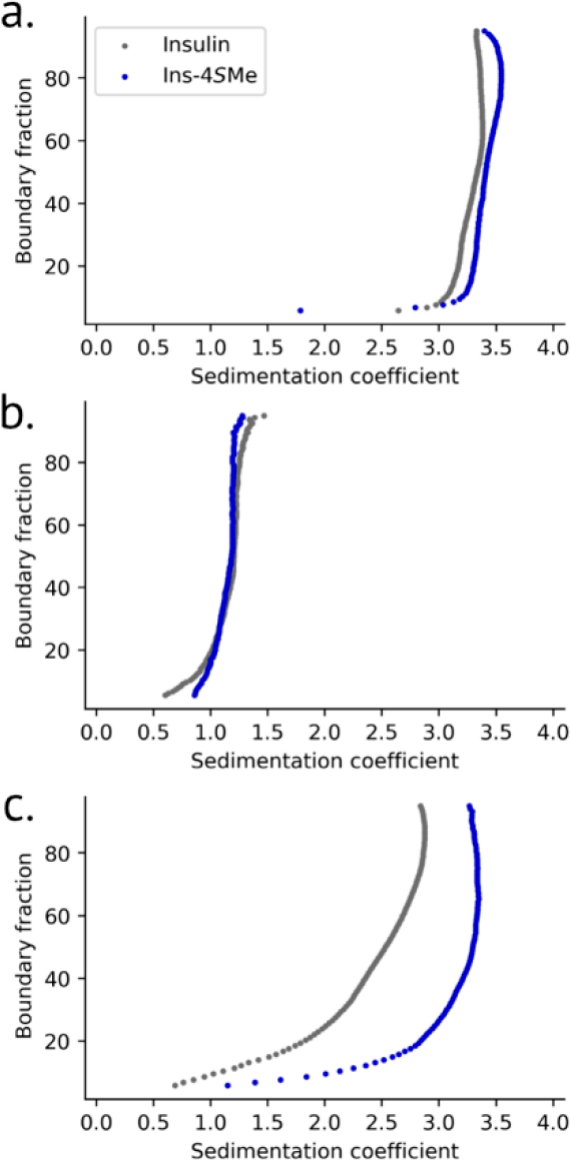
Diffusion-corrected integral sedimentation coefficient distributions. Insulin and ins-4*S*-Me were formulated under the following conditions: a. 300 μM insulin, 12.5 mM *m*-cresol, and 125 μM Zn. b. Four μM insulin, 167 μM *m*-cresol, 1.67 μM Zn. c. 300 μM insulin. Samples described in panels a and c were measured using interference optics due to high absorbance from insulin and *m*-cresol; those in panel b used absorbance at 225 nm. All samples were in 25 mM tris buffer, pH 8.0.

We examined the oligomerization of insulin and ins-4*S*-Me in more detail by sedimentation velocity analytical ultracentrifugation. At 300 μM in the presence of zinc and *m*-cresol, the sedimentation coefficients of both insulin (3.3S) and ins-4*S*Me (3.4S) correspond to the hexamer^39^ (Figure 5a, Table S5). After dilution to 4 μM, both insulins behave as monomers (Figure 5b, Table S5),^40^ further validating that the dilution kinetics measured above describe hexamer dissociation.

Interestingly, we found that at these higher concentrations, 4*S*-methylation of ProB28 affects insulin oligomerization in the absence of ligands. Compared to the broad sedimentation profile of insulin, which indicates the presence of multiple oligomers between monomer and hexamer,^41^ the sedimentation of ins-4*S*-Me (3.1S) is nearly unchanged when zinc and *m*-cresol are omitted (Figure 5c). These results are counterintuitive: when formulated with ligands, ins-4*S*-Me dissociates more rapidly upon dilution relative to insulin (Figure 4f). However, at 300 μM and in the absence of ligands, 4*S*-methylation instead stabilizes the hexamer. We note that insulin behavior depends upon its formulation;^24,39^ for instance, the crystal structure of the insulin hexamer in the presence of zinc and *m*-cresol^37^ is distinct from that under ligand-free conditions.^42^ Perhaps the effect of 4*S*-methylation on insulin oligomerization is similarly context-dependent.

### 4ene at Position B28 Accelerates Fibril Formation

**Figure 6:**
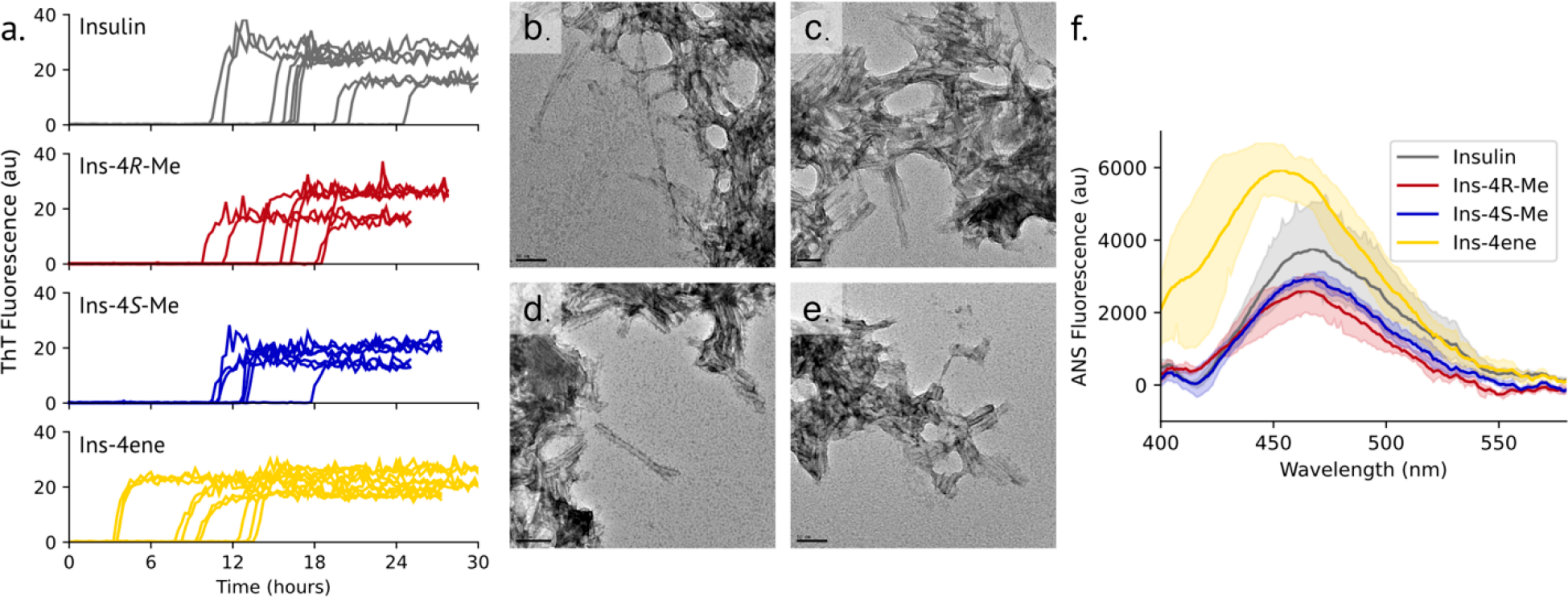
Fibrillation of insulin variants. a. Insulin variants (60 μM in 100 mM phosphate buffer, pH 8.0) were incubated at 37 °C with vigorous shaking, and fibril formation was monitored by ThT fluorescence over time. b-e. Representative TEM images of insulin (b; scale bar: 50 μm), ins-4*R*-Me (c, 50 μm), ins-4*S*-Me (d, 100 μm), and ins-4ene (e, 50 μm) aggregates. f. ANS emission spectra of insulin variants (1 μM insulin variant labeled with 5 μM ANS in 100 mM phosphate buffer, pH 8.0).

To assess stability against aggregation, insulins were subjected to vigorous shaking at 37 °C, and fibril formation was monitored over time with the fibril-specific dye thioflavin T (ThT; Figure 6a; Table S4). Under these conditions, the lag time to fibril formation for insulin was 16.6 ± 4.1 h. Introduction of either 4*R*-Me or 4*S*-Me at position B28 did not significantly affect the lag time (15.0 ± 3.5 and 12.5 ± 2.5 h, respectively). However, ins-4ene (8.2 ± 4.0 h) formed fibrils more rapidly than the other insulins examined here. All aggregates were fibrillar in nature when assessed by transmission electron microscopy (TEM). Compared to the micrometer-long fibrils described in previous reports,^43−46^ those observed here were relatively short (tens to hundreds of nanometers). Under these conditions, ins-4ene formed fibrils shorter than those of insulin, ins-4*R*-Me, or ins-4*S*-Me (Figure 6b-e).

We sought to better understand the molecular mechanism of the decreased stability of ins-4ene. An early step in the mechanism of insulin fibril formation is thought to be detachment of the C-terminus of the insulin B-chain from the core of the molecule, exposing hydrophobic residues.^46^ We probed insulin disorder with the dye 8-anilino-1-naphthalenesulfonic acid (ANS).^47^ Compared to the other insulins measured, ins-4ene exhibits a blue-shift in the emission maximum and an increase in fluorescence intensity upon labeling with ANS (Figure 6f; Table S4), suggesting an increased exposure of buried hydrophobic residues. We analyzed DMSO-solubilized ins-4ene fibrils by mass spectrometry but did not observe chemical modification of the exocyclic alkene (Figure S6).

## Conclusion

In this work, we have expanded the set of ncPro residues that can be incorporated into proteins in *E. coli* and demonstrated their utility in modifying the properties of recombinantly produced insulin. We found that the proline analogs 4*R*-Me, 4*S*-Me, and 4ene could be incorporated with high efficiencies into recombinant proinsulin. As this work was coming to completion, the incorporation of 4*R*-Me into recombinant thioredoxin was reported.^48^ In that report, incorporation of the 4*S* diastereomer could not be detected, perhaps because it interferes with thioredoxin folding and renders the protein unstable. In the work reported here, proinsulin is purified from the inclusion body fraction; therefore, any influence of ncPro replacement on protein stability during expression should be reduced. We were also able to detect low to modest levels of incorporation of other ncPro residues into proinsulin, such as 3-hydroxy analogs, 4-oxoproline, and the diazirine-containing ncPro photo-proline (Figure S1, Table S1). Engineering the prolyl-tRNA synthetase or other components of the *E. coli* translational machinery might lead to improved incorporation of these analogs.

In addition to expanding the side-chain volume and hydrophobic surface area that can be introduced into proteins at proline sites, the three ncPro residues studied here complement other translationally active proline analogs that have been used to probe the roles of proline conformation in determining the biophysical behavior of proteins. For instance, 4*R*-Me and 4*S*-Me have opposing conformational preferences relative to their more commonly used 4-fluoroproline counterparts^1,2,49^ and together have been used to validate the stereoelectronic origin of collagen stability.^7,49^ The amplitude of the pyrrolidine ring pucker of 4ene is expected to be attenuated compared to proline (Figure S7) and may approach that of the planar 3,4-dehydroproline.^50^ Furthermore, the olefin present in 4ene might be used as a chemical handle for protein modification at proline residues.^51,52^

This work also demonstrates the extent to which small molecular changes can affect the protein behavior. For instance, introducing an exocyclic alkene at B28 of insulin shortened the lag time of fibril formation. This finding, together with increased solvent-exposed hydrophobic surface area of ins-4ene as evidenced by its ANS emission spectrum, supports current models for the mechanism of insulin fibrillation.^46^ Furthermore, installing a simple methyl group at ProB28 of human insulin significantly accelerates dissociation from the insulin hexamer, behavior associated with achieving a rapid onset-of-action for monomeric FAIs.^23^ Insulin fibrillation is thought to proceed from the monomer state, and conditions that disfavor insulin oligomerization often promote physical denaturation.^46^ Notably, in the cases of Ins-4*R*-Me and Ins-4*S*-Me, rapid dissociation was achieved without accelerating fibril formation. Future work is needed to understand more fully the effect of 4-methylation on insulin oligomerization and to determine whether the faster dissociation observed for ins-4*R*-Me and ins-4*S*-Me *in vitro* results in faster onset-of-action *in vivo*.

## Methods

### Detailed Methods Are Provided in the Supporting Information (SI)

#### Proinsulin Expression, Refolding, and Purification

Proinsulins containing proline analogs were expressed in the *E. coli* strain CAG18515/pQE80_H27R-PI_proS. This is a proline auxotrophic strain which carries a plasmid for prolyl-tRNA synthetase (*proS*) overexpression and inducible expression of proinsulin. Briefly, the culture was grown in Andrew’s Magical Medium,^53^ a defined medium that contains 50 mg L^–1^ proline, until midlog phase. Cells were then washed and resuspended in a medium that lacks proline. The proline analog (0.5–1.0 mM) was added along with 0.5 M NaCl to induce osmotic stress conditions that increase cellular uptake of the proline analog. Proinsulin was expressed overnight with the addition of 1 mM IPTG. Specific expression conditions (*proS* variant, ncPro concentration) for each insulin variant are described in Table S3.

Proinsulin was isolated from the inclusion body fraction, solubilized (3 M urea and 10 mM cysteine), and refolded (10 mM CAPS, pH 10.7, 4 °C) over the course of 2–3 days. Purified, mature insulin was obtained after digestion with trypsin and carboxypeptidase B and purification by reversed-phase high-performance liquid chromatography (HPLC). Fractions were analyzed by matrix-assisted laser desorption/ionization time-of-flight mass spectrometry (MALDI-TOF MS), sodium dodecyl sulfate–polyacrylamide gel electrophoresis (SDS-PAGE), and analytical HPLC to verify sample quality and ensure ≥ 95% purity for all downstream analyses. Yields for ncPro-containing insulins ranged from 23 to 53 mg L^–1^, and mature insulin yields were approximately 15–50% of the proinsulin starting material. Insulin was expressed in Terrific Broth (TB) and similarly refolded and purified.

#### Reduction of Blood Glucose in Diabetic Mice

Adult (8 week old) male C57BL/6J mice were ordered from Jackson Laboratory (Bar Harbor, ME) and injected intraperitoneally with freshly prepared streptozotocin at 65 mg kg^–1^ day^–1^ for 3 consecutive days. Insulin-dependent diabetes was confirmed by detection of high glucose levels (200–600 mg dL^–1^) as measured with a glucometer. Insulin analogs were diluted to 100 μg mL^–1^ in formulation buffer (1.6 mg mL^–1^ m-cresol, 0.65 mg mL^–1^ phenol, 3.8 mg mL^–1^ sodium phosphate pH 7.4, 16 mg mL^–1^ glycerol, 0.8 μg mL^–1^ ZnCl_2_) and injected (35 μg kg^–1^) subcutaneously at the scruff. Blood glucose was measured over 2.5 h from blood sampled from the lateral tail vein.

#### Kinetics of Hexamer Dissociation

Insulin samples were dialyzed overnight against 28.6 mM tris buffer, pH 8.0, and then formulated as follows: 600 μM insulin, 250 μM ZnCl_2_, 25 mM *m*-cresol, 25 mM tris buffer, pH 8. To a stirred buffer solution containing 2.98 mL of 25 mM tris, pH 8.0 in a 10 mm quartz cuvette was injected 20 μL of the insulin formulation (150-fold dilution). Ellipticity was monitored at 222 nm over 120 s at 25 °C. A typical run led to a rapid drop in the CD signal as mixing occurred (∼5 s) and then a gradual rise to an equilibrium ellipticity representative of an insulin monomer. Data preceding the time point with the greatest negative ellipticity (representing the mixing time) were omitted from further analysis. Runs were discarded if the maximum change in mean residue ellipticity from equilibrium did not exceed 750 deg cm^2^ dmol^–1^, which indicated poor mixing. The remaining data were fit to a monoexponential function using Scipy (Python). The data presented here are from at least two separate insulin HPLC fractions measured on two different days.

#### Analytical Ultracentrifugation

Insulins were dialyzed against 28.6 mM tris buffer, pH 8.0 and formulated at 300 μM insulin, 12.5 mM *m*-cresol, and 125 μM ZnCl_2_, in 25 mM tris buffer. Ligand-free insulins (300 μM) and diluted insulins (4 μM insulin, 167 μM *m*-cresol, and 1.7 μM ZnCl_2_) were also prepared from the same dialysis samples. Diluted insulin conditions were identical to those after dilution in the CD dissociation kinetics experiments and were incubated at RT for at least one hour after dilution prior to analysis.

Velocity sedimentation experiments were performed at the Canadian Center for Hydrodynamics at the University of Lethbridge. 300 μM insulin samples were measured by interference optics due to the high absorbance from the protein and *m*-cresol. Diluted samples (4 μM insulin) were measured using absorbance optics at 225 nm. All data were analyzed with UltraScan III version 4.0 release 6606.^54^ Velocity data were initially fitted with the two-dimensional spectrum analysis^55^ to determine meniscus position and time- and radially invariant noise. Subsequent noise-corrected data were analyzed by the enhanced van Holde-Weischet analysis^56^ to generate diffusion-corrected integral sedimentation coefficient distributions.

#### Fibrillation Lag Time

Insulin samples (60 μM in 100 mM sodium phosphate, pH 8.0) were centrifuged at 22,000 g for 1 h at 4 °C prior to the addition of 1 μM thioflavin T (ThT). Each sample was shaken continuously in a 96-well, black, clear bottom plate at 37 °C, and fluorescence readings were recorded every 15 min (444 nm excitation, 485 nm emission). Fibrillation runs were performed on at least two separate HPLC fractions, each in triplicate or quadruplicate, on at least two different days. The growth phase of each fibrillation replicate was fit to a linear function, and fibrillation lag times were reported as the x-intercept of this fit. Fibril samples were stored at 4 °C until analysis by TEM.

#### ANS Fluorescence

Insulins (1 μM) were mixed with 5 μM ANS in 100 mM phosphate buffer, pH 8.0. Fluorescence emission spectra were measured in 1 cm quartz cuvettes at ambient temperature (350 nm excitation). Measurements for each insulin were performed in triplicate from three separate HPLC fractions. Spectra were smoothed before plotting and determining the emission maxima.

## Supporting Information Available

Extended experimental details, materials, and methods; nucleotide and amino acid sequences; incorporation efficiencies of 15 proline analogs; mass spectrometry characterization of proline analog incorporation, mature insulin variants, and solubilized insulin fibrils; representative purity assessment of insulin variants by SDS-PAGE; proinsulin expression conditions and yields; summaries of insulin variant characterization data; equilibrium circular dichroism spectra; models of insulin variant hexamers; and conformational analysis of proline analogs (PDF)
