# Supplementary material for: Incorporation of Aliphatic Proline Residues into Recombinantly Produced Insulin
Source: ACS Chem Biol. 2023 Nov 14;18(12):2574–81. doi: 10.1021/acschembio.3c00561 (PMC10728891; doi:10.1021/acschembio.3c00561)
Supplement: Supplementary file 1 — cb3c00561_si_001.pdf [file cb3c00561_si_001.pdf]

Supporting Information

Incorporation of aliphatic proline residues into recombinantly-produced insulin

Stephanie L. Breunig[1], Janine C. Quijano[2], Cecile Donohue[2], Amy Henrickson,[3] Borries Demeler,[3,4] Hsun Teresa Ku[2,5], and David A. Tirrell[1*]

[1]Division of Chemistry and Chemical Engineering, California Institute of Technology, Pasadena, California 91125, United States.
[2]Department of Translational Research and Cellular Therapeutics, Diabetes and Metabolism Research Institute, Beckman Institute City of Hope, Duarte, California 91010, United States.
[3]University of Lethbridge, Department of Chemistry and Biochemistry, Lethbridge, Alberta T1K 3M4, Canada.
[4]University of Montana, Department of Chemistry and Biochemistry, Missoula, MT 59801, United States.
[5]Irell & Manella Graduate School of Biological Science, City of Hope, Duarte, California 91010, United States.

[*]Corresponding author. Email: tirrell@caltech.edu

Table of Contents

Materials and methods S2
- Chemicals S2
- Enzymes S2
- Strains and plasmids S3
- Primers S3
- Nucleotide and amino acid sequences S3
- Screening ncPro incorporation S5
- Proinsulin expression S5
- Proinsulin refolding S6
- Insulin maturation and purification S7
- MALDI-TOF MS S7
- Circular dichroism spectroscopy S7
- Reduction of blood glucose in diabetic mice S8
- Fibrillation S8
- Transmission electron microscopy S9
- ANS fluorescence S9
- Analytical ultracentrifugation S9
- Models of ins-4*R*-Me and ins-4*S*-Me hexamers S9
- Mass spectrometry characterization of dissolved insulin fibrils S10
- Calculations of proline and proline analog conformation S10

Table S1: Incorporation of non-canonical proline residues S11
Table S2: Mass spectrometry characterization of insulin variants S12

Table S3: Expression conditions and insulin yields S13
Table S4: Summary of insulin variant characterization S14
Table S5: Sedimentation coefficients of insulin and ins-4*S*-Me...S15
Figure S1: SDS-PAGE after ncPro incorporation S16
Figure S2: Purity of insulin samples assessed by SDS-PAGE S17
Figure S3: Changes in CD signal after dilution are not due to protein denaturation S18
Figure S4: Equilibrium CD spectra after dilution..S19
Figure S5: Models of ins-4*R*-Me and ins-4*S*-Me hexamers...S20
Figure S6: Deconvoluted mass spectra of insulin and ins-4ene fibrils S21
Figure S7: Conformations of proline, 4-methyleneproline, and 3,4-dehydroproline S22
References...S23

Materials and methods

Chemicals:

All chemicals were purchased from MilliporeSigma unless otherwise indicated. 4-methyleneproline (4ene) was purchased as the N-boc protected version from Acros Organics, and deprotected with trifluoroacetic acid (TFA) in dichloromethane. 4ene was extracted with H_2O and lyophilized; complete deprotection and >95% purity were verified by ^1H NMR. All other proline analogs were used as received: 4*R*-methylproline (4*R*-Me) was purchased from Advanced Chemblocks as the hydrochloride salt; ^1H NMR analysis indicated the presence of 5-10% of the 4*S*-methylproline diastereomer. 2-Methylproline (2-Me) was purchased from Advanced ChemBlocks as the hydrochloride salt. 4*S*-Methylproline (4*S*-Me) was purchased from AstaTech as the hydrochloride salt. L-*cis*-pyrrolidine-2,4-dicarboxylic acid (4*S*-COOH) was purchased from Boc Sciences. L-*trans*-pyrrolidine-2,4-dicarboxylic acid (4*R*-COOH) was purchased from Tocris Biosciences. L-pyroglutamic acid (5-oxo) was purchased from Aldrich. 4,4-dimethylproline (44-diMe) was purchased from J&W Pharmlab. Piperazine-2-carboxylic acid (Pip-Az) was purchased from Ark Pharm as the dihydrochloride salt. (2*S*,5*S*)-5-hydroxypiperidine-2-carboxylic acid (Pip-OH) was purchased from Ark Pharm. 4*S*-aminoproline (4*S*-NH_2) was purchased from Toronto Research Chemicals as the dihydrochloride salt. 3*R*-hydroxyproline (3*R*-OH) was purchased from Combi-Blocks. 3*S*-hydroxyproline (3*S*-OH) was purchased from Ark Pharm. 4-Oxoproline (4-oxo) was purchased as the hydrobromide salt from MilliporeSigma.

Enzymes:

Restriction enzymes, kinases, and ligases were purchased from New England Biolabs. Trypsin was purchased from MilliporeSigma. Carboxypeptidase B was purchased from Worthington Biochemical. Glu-C peptidase was purchased from Promega.

Strains and plasmids:

The proline-auxotrophic *E. coli* strain CAG18515 was obtained from the Coli Genetic Stock Center (CGSC) at Yale University. Strain DH10B was used for standard cloning operations; electrocompetent CAG18515 were transformed with purified plasmid products.

The plasmid pQE80_H27R-PI_proS contains an IPTG-inducible proinsulin gene and the *E. coli* prolyl-tRNA synthetase gene controlled by its endogenous promoter. Proinsulin is translationally fused to an N-terminal leader peptide (H27R) that increases expression yields,[1] and a 10x-his tag to facilitate proinsulin enrichment after refolding. The gene for H27R-PI was ordered as a g-Block gene fragment from Integrated DNA Technologies (IDT) after codon optimization of the N-terminal leader peptide. A restriction enzyme cloning approach (XhoI and BamHI restriction enzyme cut sites) was used to replace the hexahistidine-tagged proinsulin gene in the plasmid pQE80PI-proS, which was described previously.[2] Correct installation of the gene of interest was verified by Sanger sequencing.

A blunt-end ligation approach was used to install the C443G and M157Q mutations. The *proS*-containing plasmid was amplified with primers AL01004_fwd & AL01004_rev (C443G), or AL01005_fwd & AL01005_rev2 (M157Q). The linear PCR product was phosphorylated (T4 PNK) and circularized (T4 DNA ligase). Correct installation of the point mutation was verified by Sanger sequencing.

Primers:

DNA oligos were purchased from Integrated DNA Technologies (IDT). Nucleotides responsible for installing the M157Q and C443G mutations are underlined.

AL01004_fwd: ATACCGTAGCCACCCATCGTCAGG
AL01004_rev: CGGGGTAACGCGTGTGGT
AL01005_fwd: AGCATCTTTCTGCAGGAATTCGC
AL01005_rev2: TACTCTTTCCATACTTCTCAGGAATCC

Nucleotide and amino acid sequences:

H27R-PI: The g-Block gene fragment was purchased from IDT. The coding sequence is in UPPERCASE, XhoI and BamHI cut sites are underlined.

gccctttcgtcttcacctcgagaaatcataaaaaatttatttgctttgtgagcggataacaattataatagattcaattgtgagcggataac
aatttcacacagaattcattaaagaggagaaattaactATGACAATGATCACTAATTCACCCGAGATTTCCCACCATCA
TCATCATCATCACCACCACCATCAGTTGATCTCGGAGGCCCGTTTTGTGAACCAGCACCTGTGCGGTAGC
CACCTGGTGGAAGCTCTGTACCTGGTTTGCGGTGAGCGTGGTTTCTTCTACACGCCAAAGACCCGCCGT
GAAGCTGAAGATCTGCAGGTGGGCCAGGTAGAACTGGGCGGTGGTCCGGGTGCCGGCTCTCTGCAACC
GCTGGCACTGGAAGGTTCCCTGCAAGCGCGTGGTATCGTAGAGCAGTGCTGTACTTCTATCTGCTCCCT
GTACCAGCTGGAGAACTACTGTAATTAAggatccgcatgcgagc

The sequence of the H27R leader peptide is underlined, proinsulin is in **bold**. The A-chain and B-chain in mature insulin are colored **red** and **blue**, respectively.

MTMITNSPEISHHHHHHHHHHQLISEAR**FVNQHLCGSHLVEALYLVCGERGFFYTPKTRREAEDLQVGQVE**
LGGGPGAGSLQPLALEGSLQARGIVEQCCTSICSLYQLENYCN

proS: the endogenous *proS* promoter is in **bold**, the coding sequence is underlined.

ATTCACGC**CCTTCTCTTTTGACATTTCTTTTGCACTGGTAAACTAAATCAC**TTTTTTTTGTCCCAGGCTCGC
CTTGAGCCTGTTCTACCTTCCAACTGGAACCGTAACAACATGCGTACTAGCCAATACCTGCTCTCCACTCT
CAAGGAGACACCTGCCGACGCCGAGGTGATCAGCCATCAGCTGATGCTGCGCGCCGGGATGATCCGCA
AGCTGGCCTCCGGGTTATATACCTGGCTGCCGACCGGCGTGCGCGTTCTGAAAAAAGTCGAAAACATCG
TGCGTGAAGAGATGAACAACGCCGGTGCGATCGAGGTGTCGATGCCGGTGGTTCAGCCAGCCGATTTG
TGGCAAGAGAGTGGTCGTTGGGAACAGTACGGTCCGGAACTGCTGCGTTTTGTTGACCGTGGCGAGCG
TCCGTTCGTACTCGGCCCAACTCATGAAGAAGTTATCACTGACCTGATTCGTAACGAGCTTAGCTCTTAC
AAACAGCTGCCGCTGAACTTCTATCAGATCCAGACCAAGTTCCGCGACGAAGTGCGTCCGCGTTTCGGC
GTCATGCGTTCCCGCGAATTCCTGATGAAAGATGCTTACTCTTTCCATACTTCTCAGGAATCCCTGCAGGA
AACCTACGATGCAATGTATGCGGCCTACAGCAAAATCTTCAGCCGCATGGGGCTGGATTTCCGCGCCGT
ACAAGCCGACACCGGTTCTATCGGCGGCAGCGCCTCTCACGAATTCCAGGTGCTGGCGCAGAGCGGTG
AAGACGATGTGGTCTTCTCCGACACCTCTGACTATGCAGCGAACATTGAACTGGCAGAAGCTATCGCGC
CGAAAGAACCGCGCGCTGCTGCTACCCAGGAAATGACGCTGGTTGATACGCCGAACGCGAAAACCATC
GCGGAACTGGTTGAACAGTTCAATCTGCCGATTGAGAAAACGGTTAAGACTCTGCTGGTTAAAGCGGTT
GAAGGCAGCAGCTTCCCGCAGGTTGCGCTGCTGGTGCGCGGTGATCACGAGCTGAACGAAGTTAAAGC
AGAAAAACTGCCGCAGGTTGCAAGCCCGCTGACTTTCGCGACCGAAGAAGAAATTCGTGCCGTGGTTAA
AGCCGGTCCGGGTTCACTGGGTCCGGTAAACATGCCGATTCCGGTGGTGATTGACCGTACCGTTGCGGC
GATGAGTGATTTCGCTGCTGGTGCTAACATCGATGGTAAACACTACTTCGGCATCAACTGGGATCGCGA
TGTCGCTACCCCGGAAGTTGCAGATATCCGTAACGTGGTGGCTGGCGATCCAAGCCCGGATGGCCAGG
GTAGGCTGCTGATCAAACGTGGTATCGAAGTTGGTCACATCTTCCAGCTGGGTACCAAGTACTCCGAAG
CACTGAAAGCCTCCGTACAGGGTGAAGATGGCCGTAACCAAATCCTGACGATGGGTTGCTACGGTATCG
GGGTAACGCGTGTGGTAGCTGCGGCGATTGAGCAGAACTACGACGAACGAGGCATCGTATGGCCTGAC
GCTATCGCGCCGTTCCAGGTGGCGATTCTGCCGATGAACATGCACAAATCCTTCCGCGTACAAGAGCTT
GCTGAGAAACTGTACAGCGAACTGCGTGCACAAGGTATCGAAGTGCTGCTGGATGACCGCAAAGAGCG
TCCGGGCGTGATGTTTGCTGATATGGAACTGATCGGTATTCCGCACACTATTGTGCTGGGCGACCGTAA
CCTCGACAACGACGATATCGAATATAAATATCGTCGCAACGGCGAGAAACAGTTAATTAAGACTGGTGA
CATCGTCGAATATCTGGTGAAACAGATTAAAGGCTGA

MRTSQYLLSTLKETPADAEVISHQLMLRAGMIRKLASGLYTWLPTGVRVLKKVENIVREEMNNAGAIEVSMP
VVQPADLWQESGRWEQYGPELLRFVDRGERPFVLGPTHEEVITDLIRNELSSYKQLPLNFYQIQTKFRDEVRP
RFGVMRSREFLMKDAYSFHTSQESLQETYDAMYAAYSKIFSRMGLDFRAVQADTGSIGGSASHEFQVLAQS
GEDDVVFSDTSDYAANIELAEAIAPKEPRAAATQEMTLVDTPNAKTIAELVEQFNLPIEKTVKTLLVKAVEGSS
FPQVALLVRGDHELNEVKAEKLPQVASPLTFATEEEIRAVVKAGPGSLGPVNMPIPVVIDRTVAAMSDFAAG
ANIDGKHYFGINWDRDVATPEVADIRNVVAGDPSPDGQGRLLIKRGIEVGHIFQLGTKYSEALKASVQGEDG
RNQILTMGCYGIGVTRVVAAAIEQNYDERGIVWPDAIAPFQVAILPMNMHKSFRVQELAEKLYSELRAQGIE
VLLDDRKERPGVMFADMELIGIPHTIVLGDRNLDNDDIEYKYRRNGEKQLIKTGDIVEYLVKQIKG*

Screening ncPro incorporation:

A single colony of *E. coli* strain CAG18515/pQE80_H27R-PI_proS was used to inoculate a culture of Luria Bertani (LB) medium supplemented with ampicillin. The culture was grown overnight at 37°C until stationary phase was reached, then diluted 1:100 into 100 mL of 1x M9 medium, supplemented with all twenty canonical amino acids. The medium composition of M9 is as follows: 8.5 mM NaCl, 18.7 mM NH_4Cl, 22 mM KH_2PO_4, 47.8 mM Na_2HPO_4, 0.1 mM $CaCl_2$, 1 mM $MgSO_4$, 3 mg L^{-1} $FeSO_4$, 1 µg L^{-1} trace metals [Cu^{2+}, Mn^{2+}, Zn^{2+}, MoO_4^{2-}], 35 mg L^{-1} thiamine HCl, 10 mg L^{-1} biotin, 20 mM D-glucose, 100 mg L^{-1} ampicillin, 50 mg L^{-1} of each L-amino acid.

The culture was grown at 37°C until it reached OD ~0.8, after which it was subjected to a medium shift: cells were pelleted via centrifugation (5,000 g, 5 min, 4°C) and washed twice with 10 mL ice-cold 0.9% NaCl. Washed cells were resuspended in 80 mL of 1.25x M9 –Pro, a 1.25x concentrated form of M9 that omits proline. The culture was split into 4 mL aliquots, and incubated for 30 min at 37°C to deplete residual proline. A 1 mL solution containing 2.5 mM ncPro and 1.5 M NaCl was added (0.5 mM ncPro and 0.3 M NaCl working concentrations). After 30 min of incubation at 37°C to allow for ncPro uptake, proinsulin expression was induced by the addition of 1 mM IPTG. Cultures were incubated for 2.5 h at 37°C, after which cells were harvested via centrifugation and stored at -80°C until further processing.

Cell pellets were thawed and lysed with B-PER Complete (Thermo Fisher Scientific) for 1 h at room temperature with shaking, then centrifuged (20,000 g, 10 min) and the supernatant discarded. The pellet (containing insoluble proinsulin) was washed once with Triton wash buffer (2 M urea, 20 mM Tris, 1% Triton X-100, pH 8.0), and twice with ddH_2O. The pellet was resuspended in solubilization buffer (8 M urea, 300 mM NaCl, 50 mM NaH_2PO_4, pH 8.0), and proinsulin was allowed to dissolve for 1 h at room temperature with shaking. Samples were centrifuged, and the supernatant removed for analysis by SDS-PAGE and MALDI-TOF (described in the section entitled "MALDI-TOF MS" below).

Proinsulin expression:

A single colony of *E. coli* strain CAG18515 harboring plasmid pEQ80_H27R-PI_proS (or the corresponding plasmid with a point mutation in the *proS* gene; see Table S3) was used to inoculate 70 mL of LB medium containing ampicillin, and the culture was grown overnight at 37°C to stationary phase. The overnight culture was used to inoculate 5 L (as 4x1.25 L cultures) of 1x Andrew's Magical Medium (AMM),[3] a defined medium containing all 20 proteinogenic amino acids, in 2.8 L Fernbach flasks. The composition of AMM was the following: 3.60 g L^{-1} glucose, 3.5 g L^{-1} KH_2PO_4, 6.56 g L^{-1} $K_2HPO_4{\bullet}3H_2O$, 3.5 g L^{-1} $(NH_4)_2HPO_4$, 8.37 g L^{-1} MOPS, 0.72 g L^{-1} tricine, 2.92 g L^{-1} NaCl, 0.51 g L^{-1} NH_4Cl, 0.26 g L^{-1} $MgCl_2{\bullet}7H_2O$, 50 mg L^{-1} K_2SO_4, 0.246 mg L^{-1} $MgSO_4{\bullet}7H_2O$, 12.3 mg L^{-1} $CaCl_2$,$\bullet 2H_2O$, 2.8 mg L^{-1} $FeSO_4{\bullet}7H_2O$, 0.5 mg L^{-1} thiamine, 24 µg L^{-1} boric acid, 1 µg L^{-1} trace metals (Cu^{2+}, Mn^{2+}, Zn^{2+}, MoO_4^{2-}), and 50 mg L^{-1} each amino acid.

When growth reached mid-exponential phase (OD_{600} ~0.8), the culture was subjected to a medium shift: cells were pelleted via centrifugation (5,000 g, 5 min, 4°C) and washed twice with 100 mL ice-cold 0.9% NaCl. Washed cells were resuspended in 1 L of 1.25x AMM -Pro, a 1.25x concentrated form of AMM that omits proline. Cells were incubated for 30 min at 37°C to deplete

residual proline, after which 250 mL of a solution containing 2.5-5.0 mM ncPro (see Table 4.S3) and 2.5 M NaCl was added (0.5-1.0 mM ncPro and 0.5 M NaCl working concentrations). After 30 min of incubation at 37°C to allow for ncPro uptake, proinsulin expression was induced by the addition of isopropylthio-β-galactosidase (IPTG, 1 mM). Cultures were incubated overnight at 37°C, after which cells were harvested via centrifugation and stored at -80°C until further processing.

Proline-containing proinsulin was expressed using strain CAG18515 harboring plasmid pQE80-H27R-PI_proS in 7.5 L (as 6 x 1.25 L cultures) of Terrific Broth (TB). IPTG (1 mM) was added at mid-log phase (OD_{600} ~0.8) to induce proinsulin expression. Cultures were incubated at 37°C for 3 h, after which cells were harvested via centrifugation and stored at -80°C until further processing.

Proinsulin refolding:

Cell pellets were warmed from -80°C to room temperature and resuspended in 5 mL IB buffer (50 mM tris, 100 mM NaCl, 1 mM EDTA, pH 8.0) per gram cell pellet. Lysozyme (1 mg L^{-1}) and phenylmethylsulfonyl fluoride (PMSF, 1 mM) were added, and the slurry was placed on ice for 30 min before cells were lysed via sonication. The lysate was centrifuged (14,000 g, 30 min, 4°C) and the soluble fraction was discarded. The pellet was washed twice with IB buffer + 1% Triton X-100, once with IB buffer, and once with water; this final step required centrifugation for 45 min. The washed inclusion body pellet was resuspended in a minimal amount of water, and the mass of proinsulin in the inclusion body pellet was estimated by SDS-PAGE.

In preparation for proinsulin refolding, the inclusion body was resuspended in 3 M urea and 10 mM cysteine in water, such that the proinsulin concentration was 1 mg proinsulin per L total slurry. To dissolve proinsulin, the pH was adjusted to 12 and sample stirred for 1 h at room temperature. At this stage, ncPro incorporation was assessed by MALDI-TOF, as described in the section entitled "MALDI-TOF MS" below. The solubilized proinsulin solution was diluted ten-fold into refolding buffer (10 mM CAPS, pH 10.6) that had been pre-cooled to 4°C. The pH of the refolding solution was adjusted to 10.7 and the sample stored at 4°C; care was taken to ensure that the solution pH remained between 10.6 and 10.8 throughout the refolding process. Proinsulin refolding progress was monitored by reverse-phase HPLC, and usually reached completion within 50 h.

Proinsulin was enriched from the refolding solution after adjusting the pH to 8.0 and incubating the sample overnight with Ni-NTA resin and 10 mM imidazole. The resin was washed with wash buffer (25 mM imidazole in PBS, pH 8.0), and proinsulin was eluted with elution buffer (250 mM imidazole in PBS, pH 8.0). Fractions containing proinsulin were combined and extensively dialyzed against 10 mM sodium phosphate, pH 8.0.

Insulin maturation and purification:

Refolded proinsulin was warmed to 37°C and digested with trypsin (20 U mL^{-1}) and carboxypeptidase-B (10 U mL^{-1}) at 37°C for 90 min to remove the N-terminal tag and C-chain. Digestion was halted by adjusting the pH to ~3 with 6 N HCl.

Insulins were immediately purified after proteolysis by reverse-phase HPLC on a C_4 column (Penomenex Jupiter 5 µm particle size, 300 Å pore size, 250x10 mm) using 0.1% TFA in water (solvent A) and 0.1% TFA in acetonitrile (solvent B) as mobile phases. A gradient of 25-32% solvent B was applied over 65 min, and fractions containing insulin were collected. Samples for purity analysis were removed at this stage; the remaining portion of the fraction was lyophilized. Each insulin fraction was analyzed by analytical reverse-phase HPLC, MALDI-TOF MS (Figure 2e-h), and SDS-PAGE (Figure S2) to verify sample quality and ensure ≥95% purity for all downstream analyses. Lyophilized insulin powders were stored at -20°C until further use.

MALDI-TOF MS:

To assess levels of incorporation of ncPros into the corresponding proinsulins, samples were digested with Glu-C, which yields a peptide fragment containing ProB28 (^{50}RGFFYT**P**KTRRE). A 20 µL aliquot of proinsulin was subjected to cysteine reduction (5 mM DTT, 55°C for 20 min) and alkylation (15 mM iodoacetamide, room temperature for 15 min in the dark), prior to 10-fold dilution into 100 mM NH_4HCO_3, pH 8.0 (100 µL final volume). Digestion was started with addition of 0.6 µL Glu-C (0.5 µg μL^{-1} in ddH_20) at 37°C for 2.5 h. The digestion reaction was quenched by adding 10 µL of 5% TFA. Peptides were desalted using ZipTip C_{18} columns (MilliporeSigma) according to the manufacturer's protocol. Desalted peptides (in 50% acetonitrile, ACN; 0.1% TFA) were diluted 3:1 into the matrix solution (α-cyanohydroxycinnamic acid in 50% ACN, 0.1% TFA) and analyzed by MALDI-TOF MS. Analog incorporation was calculated by comparing the area under the curve (AUC) of the ncPro form of the peptide (m/z = 1572 for 4*R*-Me and 4*S*-Me, and 1570 for 4ene) with the AUC of the canonical proline peptide (m/z = 1558).

HPLC-purified insulins were analyzed as full-length, mature proteins. Aliquots directly from HPLC purification (~30% ACN, 0.1% TFA) were mixed 1:1 with matrix solution (sinapic acid in 30% ACN, 0.1% TFA) before analysis by MALDI-TOF MS.

Circular dichroism spectroscopy:

Equilibrium measurements: The circular dichroism spectra of insulin samples (60 µM in 100 mM sodium phosphate, pH 8.0) were measured at 25°C in 1 mm quartz cuvettes on an Aviv Model 430 Circular Dichroism Spectrophotometer using a step size of 0.5 nm and averaging time of 1 s. A reference buffer spectrum was subtracted from each sample spectrum.

Kinetic measurements: Insulin samples in 100 mM sodium phosphate buffer pH 8.0 were dialyzed overnight against 28.6 mM tris buffer, pH 8.0 (Slide-A-Lyzer dialysis cassettes, 3.5 kDa MWCO, ThermoFisher). Insulins were formulated as follows: 600 µM insulin, 250 µM $ZnCl_2$, 25 mM m-cresol, 25 mM tris buffer, pH 8. To a stirred buffer solution containing 2.98 mL of 25 mM tris, pH 8.0 in a 10 mm quartz cuvette was injected 20 µL of the insulin formulation (150-fold dilution).

Ellipticity was monitored at 222 nm over 120 s (1 s kinetic interval, 0.5 s time constant, 10 nm bandwidth) at 25°C. A typical run led to a rapid drop in CD signal as mixing occurred (~5 s), then a gradual rise to an equilibrium ellipticity representative of an insulin monomer. Data preceding the timepoint with the greatest negative ellipticity (representing the mixing time) were omitted from further analysis. Runs were discarded if the maximum change in mean residue ellipticity from equilibrium did not exceed 750 deg cm^2 $dmol^{-1}$, which indicated poor mixing. The remaining data were fit to a mono-exponential function using Scipy (Python). The data presented here are from at least two separate insulin HPLC fractions, measured on two different days.

For quality control, an equilibrium spectrum for each protein was obtained after dilution as described above; all spectra approached that of the insulin monomer[4] (Figure S4). The CD spectrum of human insulin under pre-dilution formulation conditions was obtained using a 0.1 mm quartz cuvette. In each case, a blank spectrum containing all buffers and ligands was subtracted from the sample spectrum.

Reduction of blood glucose in diabetic mice:

Adult (8 week old) male C57BL/6J mice were ordered from Jackson Laboratory (Bar Harbor, ME). Mice were maintained under specific pathogen-free conditions, and experiments were conducted according to procedures approved by the Institutional Animal Care and Use Committee (IACUC) at the City of Hope. Adult (8-12 week old) male mice were injected intraperitoneally (65 mg kg^{-1} day^{-1} for 3 consecutive days) with freshly prepared streptozotocin (STZ) in 50 mM citrate buffer, pH 4.5 to induce diabetes. Insulin-dependent diabetes was confirmed 3 weeks after the last dose of STZ by detection of high glucose levels (200-600 mg dL^{-1}) as measured by a glucomonitor (Freestyle, Abbott Diabetes Care, Alameda, CA) in blood sampled from the lateral tail vein. Insulin analogs were diluted to 100 μg mL^{-1} in formulation buffer (1.6 mg mL^{-1} *m*-cresol, 0.65 mg mL^{-1} phenol, 3.8 mg mL^{-1} sodium phosphate pH 7.4, 16 mg mL^{-1} glycerol, 0.8 μg mL^{-1} $ZnCl_2$). Insulin analogs were injected (35 μg kg^{-1}) subcutaneously at the scruff of diabetic 12-13 week old C57BL/6J mice. Blood glucose was measured at 0, 10, 20, 30, 40, 50, 60, 80, 100, 120, and 150 min.

Fibrillation:

Insulin samples (60 μM in 100 mM sodium phosphate, pH 8.0) were centrifuged at 22,000 g for 1 h at 4°C, prior to the addition of 1 μM thioflavin T (ThT). Each insulin (200 μL) was added to a 96-well, black, clear bottom plate (Greiner Bio-One) and sealed. Samples were shaken continuously at 960 rpm on a Varioskan multimode plate reader at 37°C, and fluorescence readings were recorded every 15 min (444 nm excitation, 485 nm emission). Fibrillation runs were performed on at least two separate HPLC fractions, each in triplicate or quadruplicate, on at least two different days. The growth phase of each fibrillation replicate was fit to a linear function, and fibrillation lag times were reported as the x-intercept of this fit. Fibril samples were stored at 4°C until analysis by TEM and mass spectrometry.

Transmission electron microscopy:

Insulin fibrils were centrifuged (5,000 g, 1 min), then washed twice and resuspended in ddH_2O. Fibrils were stained with 2% uranyl acetate on a 300-mesh formvar/carbon coated copper grid (Electron Microscopy Sciences) and imaged on a Tecnai T12 LaB6 120 eV transmission electron microscope.

ANS fluorescence:

Insulins (1 µM) were mixed with 5 µM ANS in 100 mM phosphate buffer, pH 8.0. Fluorescence emission spectra were measured in 1 cm quartz cuvettes at ambient temperature using a PTI QuantaMaster fluorescence spectrofluorometer. A 350 nm excitation wavelength and scan rate of 2 nm s^{-1} were used. Measurements for each insulin were performed in triplicate from three separate HPLC fractions. Spectra were smoothed before plotting and determining the emission maxima.

Analytical ultracentrifugation:

Insulins were dialyzed against 28.6 mM tris buffer, pH 8.0, and formulated at 300 µM insulin, 12.5 mM *m*-cresol, and 125 µM $ZnCl_2$. Ligand-free insulins were formulated from the same dialysis sample. The insulin samples were then diluted 75-fold into 25 mM tris buffer, pH 8.0 (4 µM insulin, 167 µM *m*-cresol, 1.7 µM $ZnCl_2$), conditions identical to those after dilution in the CD dissociation kinetics experiments. Diluted insulins were incubated at room temperature for at least one hour after dilution prior to analysis.

Velocity sedimentation experiments were performed at the Canadian Center for Hydrodynamics at the University of Lethbridge. 300 µM insulin samples were measured by interference optics, due to the high absorbance from the protein and *m*-cresol; they were measured in 3 mm titanium centerpieces from Nanolytics, fitted in a standard Beckman Coulter cell housing using a 3mm spacer above and below the centerpiece. Diluted samples (4 µM insulin) were measured using absorbance optics at 225 nm in standard Beckman Coulter 1.2 epon-charocol centerpieces. All samples were measured at 50,000 RPM and 20°C in standard Beckman Coulter cell housings fitted with a 1.2 cm epon-charcoal centerpiece and sapphire windows. All data were analyzed with UltraScan III version 4.0 release 6606.[5] Velocity data were initially fitted with the two-dimensional spectrum analysis[6] to determine meniscus position and time- and radially-invariant noise. Subsequent noise-corrected data were analyzed by the enhanced van Holde-Weischet analysis[7] to generate diffusion-corrected integral sedimentation coefficient distributions.

Models of ins-4*R*-Me and ins-4*S*-Me hexamers:

Crystal structures of the T_6 (PDB: 1MSO) and R_6 (1EV6) insulin hexamers were downloaded from the Protein Data Bank and visualized with Pymol. The hydrogen atoms at the C^{γ} position of ProB28 were replaced with methyl groups; no additional energy minimization was used.

Mass spectrometry characterization of dissolved insulin fibrils:

Samples containing insulin fibrils were centrifuged (5,000 g, 1 min, 4°C), washed twice with ddH_2O and dissolved in dimethylsulfoxide (DMSO). Dissolved fibrils were reduced (5 µM DTT, 55°C, 20 min), then diluted ten-fold into MS loading buffer (2% ACN, 0.2% formaic acid, FA, in water). 8 µL of this sample was injected onto a Thermo EASY-Spray column (ES902, C18, 2um, 100A, 75 um x 25 cm) equipped with an Acclaim PepMap trapping column (C18, 3um, 100A, 75 um x 2 cm), and analyzed using a Thermo Orbitrap Eclipse Tribrid Mass Spectrometer coupled with a Thermo Easy nLC-1200. The resulting raw files were deconvoluted using MASH Explorer.[8]

Calculations of proline and proline analog conformation:

The equilibrium geometry conformations of the N-methyl, O-methyl ester protected versions of proline, 4-methyleneproline, and 3,4-dehydroproline in water were calculated using Spartan Student (Wavefunction) at the B3LYP/G-31+G** level of theory. Pseudorotation parameters were calculated from the dihedral angles about the pyrrolidine ring, as previously reported.[9]

Table S1. Incorporation of non-canonical proline residues into recombinant proinsulin.

ncPro	Expected m/z	Observed m/z‡	Expected Δ m/z#	Observed Δ m/z‡#	Incorporation efficiency‡
Proline	1557.90	1557.19 ± 0.02	–	–	–
2-Me	1571.91	n.d.*	14.01	–	–
3*R*-OH	1573.88	1573.184 ± 0.006	15.98	15.5 ± 0.7	0.672 ± 0.004
3*S*-OH	1573.88	1573.184 ± 0.008	15.98	15.990 ± 0.005	0.537 ± 0.002
4*S*-NH_2	1572.90	1572.191 ± 0.001	15.00	14.990 ± 0.006	0.17 ± 0.03
4*R*-COOH	1601.89	n.d.	43.99	–	–
4*S*-COOH	1601.89	n.d.	43.99	–	–
4-oxo	1571.87	1571.182 ± 0.009	13.97	13.974 ± 0.009	0.15 ± 0.01
4-ene	1569.89	1569.218 ± 0.008	11.99	11.971 ± 0.001	0.926 ± 0.002
4*R*-Me	1571.91	1571.241 ± 0.006	14.01	13.90 ± 0.02	0.853 ± 0.004
4*S*-Me	1571.91	1571.26 ± 0.01	14.01	14.017 ± 0.004	0.775 ± 0.007
44-diMe	1585.93	n.d.	28.03	–	–
5-oxo	1571.87	n.d.	13.97	–	–
Pip-OH	1587.91	n.d.	30.01	–	–
Pip-Az	1572.90	n.d.	15.00	–	–
Photo-pro%	528.61	528.61	8.66	8.67	0.373

‡Average ± standard deviation of two technical replicates

#Mass shift compared to the proline-containing peptide present in the spectrum

*n.d., not detected

%Digested photo-proline peptide was analyzed by LC-ESI-MS, due to diazirine photolysis during MALDI-TOF analysis. We quantified the $[M+3H]^{+3}$ ion for the proline and ncPro-containing peptides. We also note the presence (27%; m/z = 519.3) of an ion corresponding to replacement of proline by 3,4-dehydroproline (dhp). The incorporation efficiency for photo-pro reported here is with respect to the proline and dhp ions:

$$\text{photo-pro incorporation efficiency} = \frac{AUC_{photo-pro}}{AUC_{pro}+AUC_{dhp}+AUC_{photo-pro}}$$

Table S2. Mass spectrometry characterization of insulin variants.

	Digested peptide			Mature insulin	
Protein	Expected m/z	Observed m/z‡	Incorporation efficiency‡	Expected m/z	Observed m/z‡
Insulin	1557.78	1557.43 ± 0.06	–	5808.6	5807.7 ± 0.4
Ins-4*R*-Me	1571.86	1571.83 ± 0.03	0.901 ± 0.010	5822.6	5822.2 ± 0.3
Ins-4*S*-Me	1571.86	1571.70 ± 0.03	0.896 ± 0.002	5822.6	5822.34 ± 0.01
Ins-4ene	1569.84	1569.56 ± 0.02	0.9417 ± 0.0005	5820.6	5821.2 ± 0.3

‡Average ± standard deviation of two technical replicates

Table S3. Expression conditions and insulin yields.

Protein	ProRS variant	[ncPro] (mM)	[NaCl] (M)	Approx. proinsulin yield (mg L^{-1})‡	Approx. mature insulin yield (mg L^{-1})
Insulin*	wt	–	–	35	8.0
Ins-4*R*-Me	C443G	0.5	0.5	29	3.9
Ins-4*S*-Me	wt	0.5	0.5	53	7.9
Ins-4ene	M157Q	1.0	0.5	23	1.5

‡Yields determined by measuring absorbance (280 nm) after proinsulin refolding and Ni-NTA enrichment.

*Expressed in terrific broth (TB)

Table S4. Summary of insulin variant characterization.

Protein	Ellipticity ratio (208/222 nm)[‡]	Fibrillation lag time (h)[‡]	Hexamer dissociation $t_{1/2}$ (s)[*]	ANS emission maximum (nm)[‡]
Insulin	1.24 ± 0.03	16.6 ± 4.1	18.4 ± 2.8	470 ± 5
Ins-4*R*-Me	1.37 ± 0.05	15.0 ± 3.5	9.8 ± 1.8	465 ± 3
Ins-4*S*-Me	1.19 ± 0.06	12.5 ± 2.5	9.9 ± 2.5	466 ± 6
Ine-4ene	1.32 ± 0.05	8.2 ± 4.0	17.0 ± 2.3	450 ± 11

[‡]60 µM insulin, 100 mM phosphate, pH 8.0

[*]150-fold dilution of 600 µM insulin, 250 µM $ZnCl_2$, 25 mM *m*-cresol, 25 mM tris, pH 8.0

Table S5. Sedimentation coefficients of insulin and ins-4*S*-Me.

Protein	Formulation*	Dilution‡	Ligand-free%
Insulin	3.3S	1.1S	2.4S
Ins-4*S*-Me	3.4S	1.1S	3.1S

*300 µM insulin, 125 µM $ZnCl_2$, 12.5 mM *m*-cresol, 25 mM tris, pH 8.0

‡4 µM insulin, 1.7 µM $ZnCl_2$, 167 µM *m*-cresol, 25 mM tris, pH 8.0

%300 µM insulin, 25 mM tris, pH 8.0

Figure S1. SDS-PAGE analysis of proinsulin expression in media supplemented with non-canonical proline analogs. Proinsulin (12.7 kDa) was expressed after a medium shift to ncPro-containing medium. The inclusion body fraction was isolated, solubilized, and analyzed by SDS-PAGE.

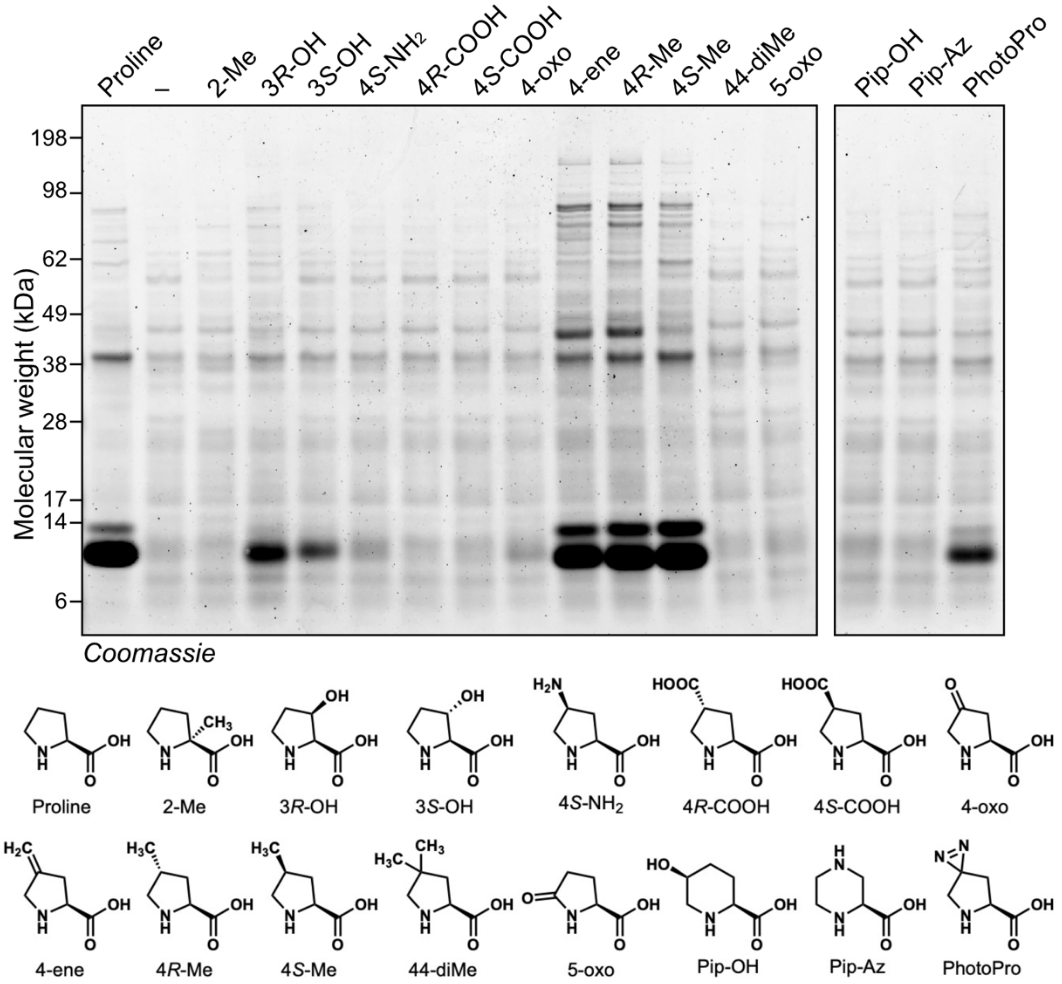

Figure S2. Purity of insulin samples assessed by SDS-PAGE. HPLC-purified insulin (a), Ins-4*R*-Me (b), Ins-4*S*-Me (c), and Ins-4ene (d) were analyzed by SDS-PAGE to validate purity; shown are representative lanes corresponding to individual HPLC fractions.

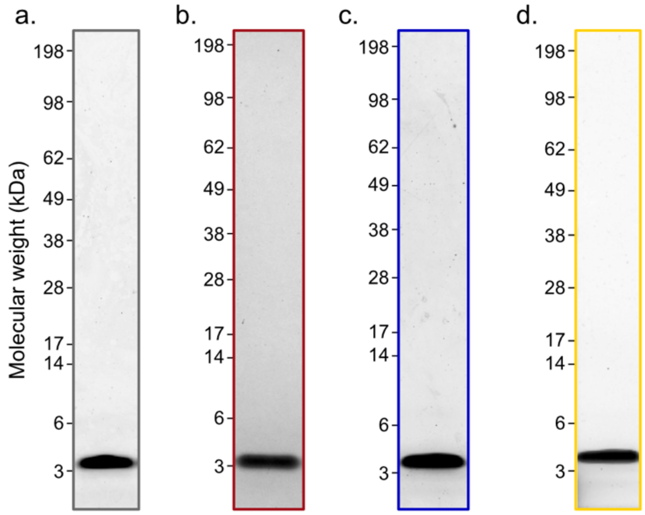

Figure S3. Changes in CD signal after dilution are not due to protein denaturation. At 60 µM, insulin is expected to exist as a dimer at pH 8, as a monomer in 20% ethanol, and in denatured form in 8 M guanidinium chloride. These spectra are overlaid with equilibrium spectra collected before and after dilution for kinetic CD measurements. Spectra below 210-215 nm were omitted for some samples due to high levels of buffer absorbance at these wavelengths.

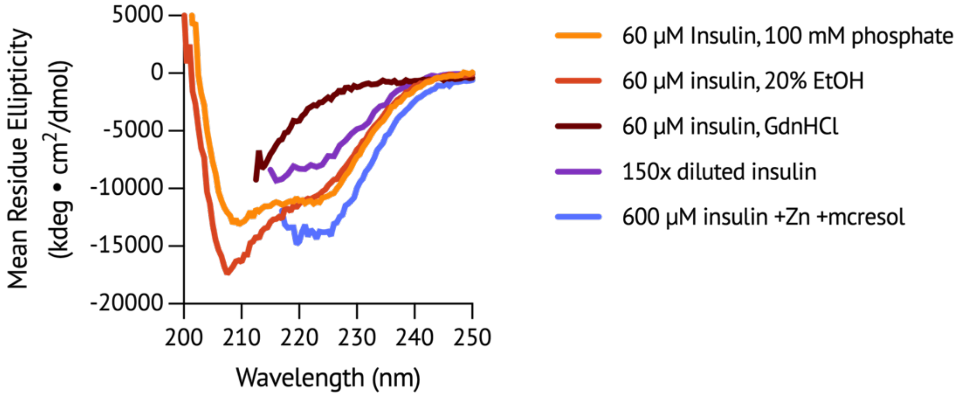

Figure S4. Equilibrium CD spectra after dilution. Equilibrium far-UV spectra of insulin (a), Ins-4*R*-Me (b), Ins-4*S*-Me (c), and Ins-4ene (d) after 150-fold dilution from the hexamer formulation. Conditions after dilution from the hexamer formulation are as follows: 4 µM insulin variant, 167 µM *m*-cresol, 1.67 µM $ZnCl_2$, 25 mM tris buffer, pH 8.0.

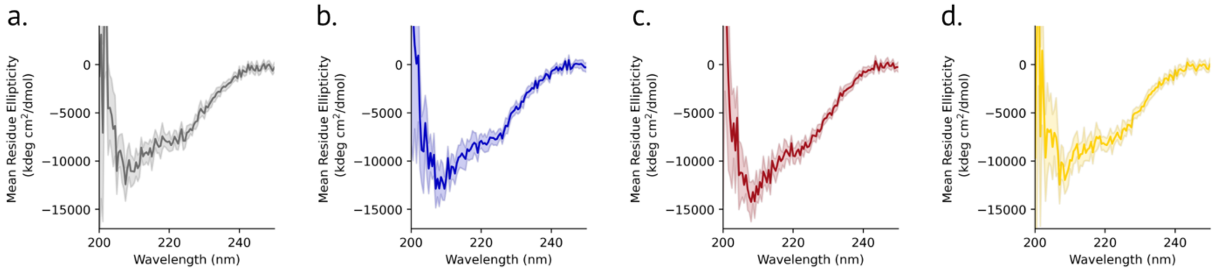

Figure S5. Models of ins-4R-Me and ins-4S-Me hexamers. 4*S*-Me and 4*R*-Me were modeled in the structures of the R_6 (**a**) and T_6 (**b**) insulin hexamers (PDB ID: 1EV3 & 1MSO, respectively). Atoms near to each methyl substituent are indicated; distance measurements are in Å.

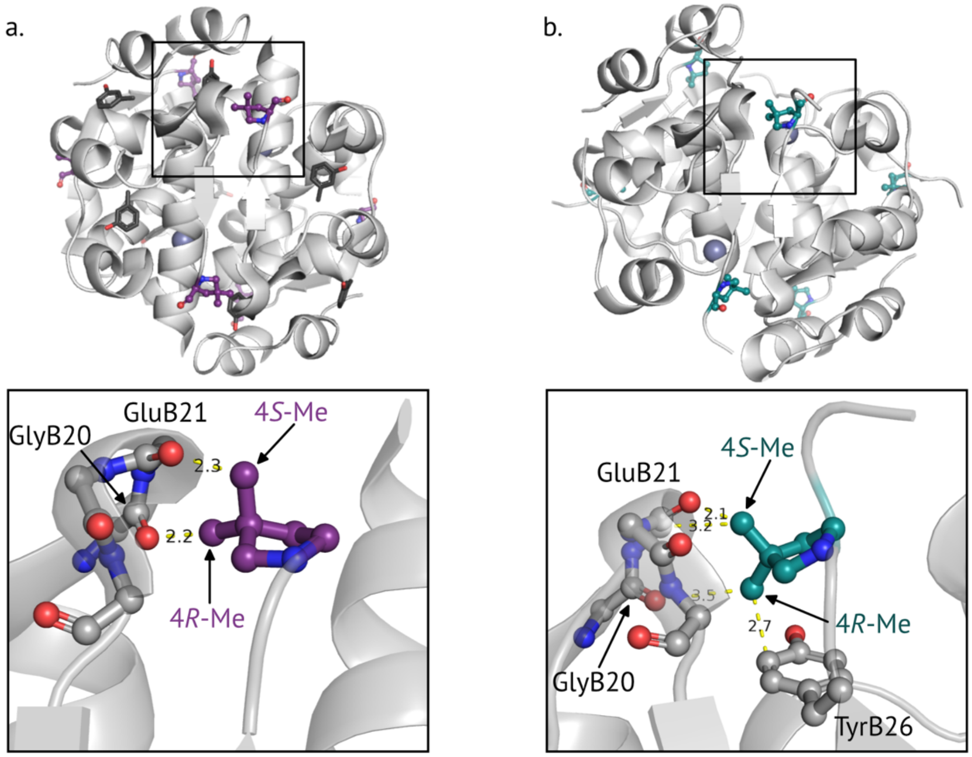

Figure S6. Deconvoluted mass spectra of insulin and ins-4ene fibrils. Insulin fibrils were dissolved with DMSO, reduced, and analyzed by mass spectrometry. Shown are the peaks corresponding to the insulin B-chain (expected molecular weights for insulin and ins-4ene: 3427.68 and 3439.68 Da). The peak present at 3464.7 Da in both samples corresponds to sample contamination by human keratin. Compared to insulin, additional peaks corresponding to chemical modification of the ins-4ene B-chain were not observed.

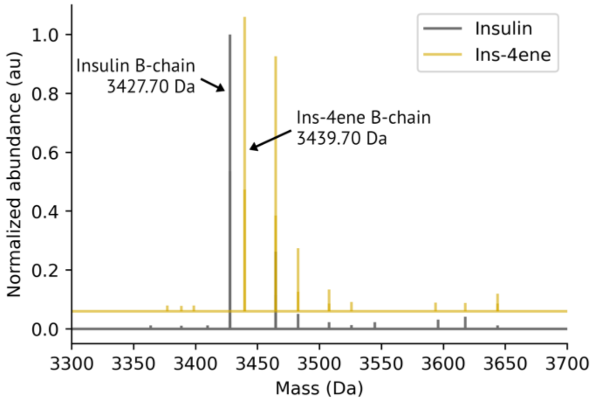

Figure S7. Conformations of proline, 4-methyleneproline, and 3,4-dehydroproline. The equilibrium geometry conformations of protected versions of proline (**a**), 4ene (**b**), and 3,4-dehydroproline (**c**) were calculated (B3LYP/G-31+G**). The pseudorotation parameters amplitude (A) and phase angle (P)[10] are indicated for each structure. Amplitude corresponds to the degree of puckering for each proline, and phase angle represents puckering geometry. The *endo* (P~198°) and *exo* (P~18°) ring puckers of proline are nearly isoenergetic and rapidly interconvert.[9] More notable in this case is the puckering amplitude, which decreases with the addition of sp^2 hybridized carbon atoms.

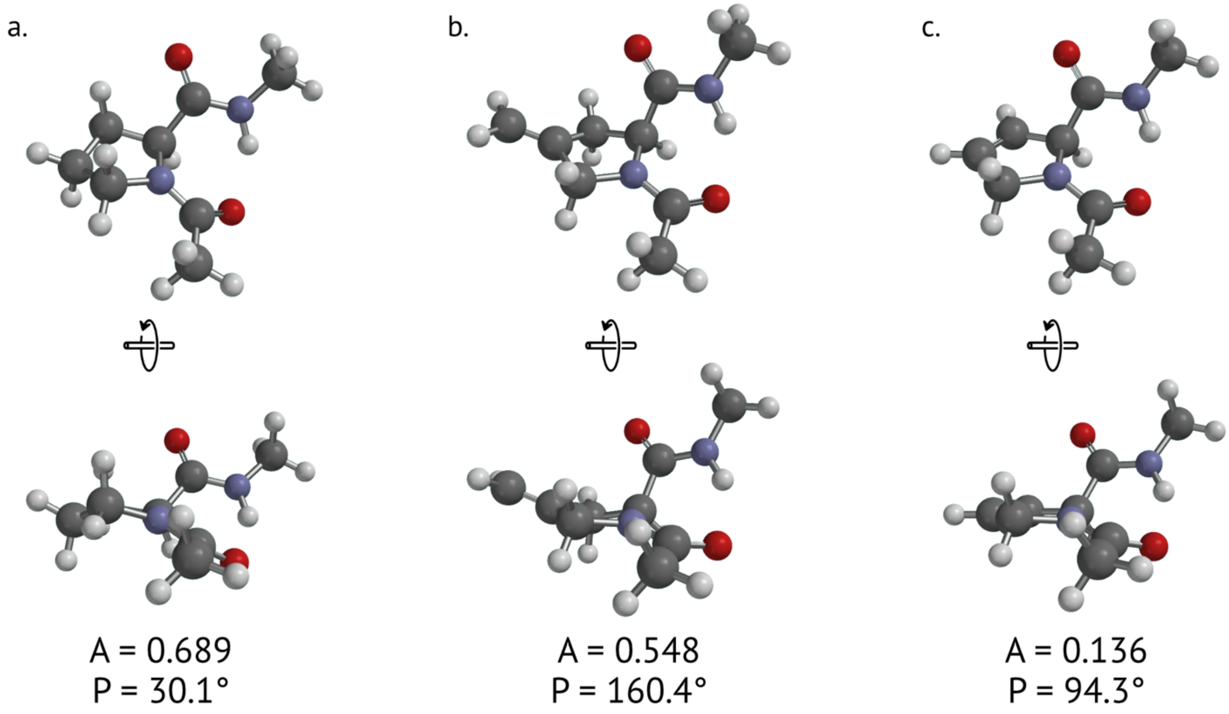

References

(1) Min, C. K.; Son, Y. J.; Kim, C. K.; Park, S. J.; Lee, J. W. Increased Expression, Folding and Enzyme Reaction Rate of Recombinant Human Insulin by Selecting Appropriate Leader Peptide. *J Biotechnol* **2011**, *151* (4), 350–356. https://doi.org/10.1016/j.jbiotec.2010.12.023.

(2) Lieblich, S. A.; Fang, K. Y.; Cahn, J. K. B.; Rawson, J.; LeBon, J.; Teresa Ku, H.; Tirrell, D. A. 4S-Hydroxylation of Insulin at ProB28 Accelerates Hexamer Dissociation and Delays Fibrillation. *J. Am. Chem. Soc.* **2017**, *139*, 8384–8387. https://doi.org/10.1021/jacs.7b00794.

(3) He, W.; Fu, L.; Li, G.; Andrew Jones, J.; Linhardt, R. J.; Koffas, M. Production of Chondroitin in Metabolically Engineered E. Coli. *Metab Eng* **2015**, *27*, 92–100. https://doi.org/10.1016/J.YMBEN.2014.11.003.

(4) Pocker, Y.; Biswas, S. B. Conformational Dynamics of Insulin in Solution. Circular Dichroic Studies. *Biochemistry* **1980**, *19* (22), 5043–5049. https://doi.org/10.1021/bi00563a017.

(5) Demeler, B.; Gorbet, G. E. Analytical Ultracentrifugation Data Analysis with Ultrascan-III. *Analytical Ultracentrifugation: Instrumentation, Software, and Applications* **2016**, 119–143. https://doi.org/10.1007/978-4-431-55985-6_8.

(6) Brookes, E.; Cao, W.; Demeler, B. A Two-Dimensional Spectrum Analysis for Sedimentation Velocity Experiments of Mixtures with Heterogeneity in Molecular Weight and Shape. *European Biophysics Journal* **2010**, *39* (3), 405–414. https://doi.org/10.1007/s00249-009-0413-5.

(7) Demeler, B.; Van Holde, K. E. Sedimentation Velocity Analysis of Highly Heterogeneous Systems. *Anal Biochem* **2004**, *335* (2), 279–288. https://doi.org/10.1016/J.AB.2004.08.039.

(8) Wu, Z.; Roberts, D. S.; Melby, J. A.; Wenger, K.; Wetzel, M.; Gu, Y.; Ramanathan, S. G.; Bayne, E. F.; Liu, X.; Sun, R.; Ong, I. M.; McIlwain, S. J.; Ge, Y. MASH Explorer: A Universal Software Environment for Top-Down Proteomics. *J Proteome Res* **2020**, *19* (9), 3867–3876. https://doi.org/https://doi.org/10.1021/acs.jproteome.0c00469.

(9) Park, H. S.; Kang, Y. K. Puckering Transition of the Proline Residue along the Pseudorotational Path: Revisited. *New Journal of Chemistry* **2021**, *45* (22), 9780–9793. https://doi.org/10.1039/D1NJ01361K.

(10) Hudáky, I.; Baldoni, H. A.; Perczel, A. Peptide Models XXXVIII. Proline Conformers from X-Ray Crystallographic Database and from Ab Initio Computations. *Journal of Molecular Structure: THEOCHEM* **2002**, *582* (1–3), 233–249. https://doi.org/10.1016/S0166-1280(01)00796-5.
